# Beyond Access: Can a School Health Initiative Facilitate Healthcare Services Utilisation for School-Going Children?

**DOI:** 10.3390/ijerph20156448

**Published:** 2023-07-26

**Authors:** Gbotemi Bukola Babatunde, Olagoke Akintola

**Affiliations:** 1 School of Public Health, Faculty of Community and Health Sciences, University of the Western Cape, Cape Town 7535, South Africa; oakintola@uwc.ac.za; 2 Graduate School of Professional Psychology, University of Denver, Denver, CO 80208, USA

**Keywords:** access, caregivers, school-going children, school-based healthcare services, school health programme, integrated school health policy, low-resource communities, South Africa

## Abstract

Accessing quality healthcare services is critical to addressing the different health challenges confronting school-going children, especially those in low-resource communities. However, the evidence of access to services is utilisation and not the mere availability of such services. This study explored caregivers’ descriptions of the factors influencing the access and utilisation of quality healthcare services for school-going children and their perceptions of the services provided through the integrated school health programme in South Africa. Qualitative interviews were conducted with 17 caregivers of school-going children in three low-resource communities of KwaZulu-Natal province. The data was analysed using thematic analysis, and the themes were clustered using components of the Aday and Andersen framework for access. Despite the efforts to expand the coverage and range of services provided through the Integrated School Health Programme (ISHP), we identified various factors that undermine the overall aim of the ISHP. Financial constraints, distance to health facilities, poor communication and information dissemination systems, low literacy levels, healthcare workers’ negative attitudes, and long waiting periods at the referral sites constitute barriers to service utilisation. Specific attention should be paid to improving the communication system between the school-health team and the caregivers, providing support for transportation, improving the attitude of the clinic staff, and providing follow-up services for children referred for further screening and treatment.

## 1. Background

Access is a central construct for assessing the performance of healthcare systems around the world [1,2,3]. Gulliford and Morgan define access as the characteristics of factors influencing the entry and use of the healthcare system [4]. It describes the opportunity or ease with which individuals can use quality services to meet their needs [2].

The evidence of access to services is utilisation and not the mere availability of such services [4,5]. Individuals in need of healthcare services may have access to the healthcare system and yet encounter difficulties in utilising the required services [6]. Hence, one could evaluate issues relating to access by examining the extent to which individuals use healthcare services [6]. This is often influenced by various factors at the individual, community, and health system levels [6]. Regardless of the efforts to improve access, people are at an increased risk of severe illness if service utilisation is low [2,4].

South Africa’s history is characterised by socio-economic, geographical, and racial inequality, which continues to challenge the success of several health initiatives aimed at promoting adequate access to quality healthcare services [7]. One such initiative that focuses on providing healthcare services for school-going children is the Integrated School Health Programme (ISHP), jointly developed by the Department of Basic Education, the Department of Health, and the Department of Social Development in South Africa in 2012 [8]. Since children spend most of their formative years in school, the ISHP provides comprehensive health packages designed to meet the health needs of school-going children [9]. The programme is being implemented across nine provinces, starting with schools in the most disadvantaged communities. The school health teams are made up of professional and enrolled nurses who provide healthcare services, including (i) screening and assessment; (ii) on-site services such as de-worming, immunization, treatment of minor ailments, and provision of health education; and (iii) referral and follow-up services [8].

Globally, studies have shown that school-based healthcare services (SBHCs) improve access to healthcare services for school-going children through the range of services offered [10,11]. Furthermore, studies conducted to evaluate the ISHP implementation in South Africa have revealed that school health services enhance school-going children’s access to: screening for different conditions, sexual and reproductive health services, HIV testing and counselling, and psychosocial services [12,13,14]. However, the literature on children’s utilisation of health services in the public healthcare facilities following referrals from school health services is sparse.

One population that has a potentially critical role to play in the ISHP are parents and other caregivers of children. Caregivers play significant roles in accessing healthcare services for their children [15]. The effective management of a child’s health condition requires the caregivers’ timely detection, decision to seek help, and the appropriate utilisation of healthcare services [15]. However, there is little attention in the literature on caregivers’ perspectives about their children’s experiences in accessing and utilising on-site (in-school) healthcare services within the ISHP. Even less is to be found about caregivers’ experiences of accessing health services in the public healthcare system following referrals from the healthcare providers of the in-school health services; our search did not yield any study on these themes. In this paper, we present the findings of a qualitative study conducted to explore caregivers’ perspectives of the on-site (in-school) health services that their primary-school-going children received through the integrated school health programme. In addition, we describe the caregivers’ perspectives about accessing follow-up services for their children in the public healthcare facilities. The findings of this study could help inform interventions to strengthen the school health initiative.

## 2. Materials and Methods

This study was carried out in three low-resource communities located within the EThekwini district of the KwaZulu-Natal province. These communities are characterised by high unemployment rates [16]. Most people are unable to find work within the community. Some, however, have to travel to urban communities to look for jobs in the service, domestic care, and gardening industries, among others. Therefore, for majority of the community members, their main income comes from government social grants [16].

Schools in these communities fall within the Quintile 1–3 categories. Government schools in South Africa are categorised into five quintiles to ensure the appropriate allocation of funds and other resources. Schools situated in the most economically disadvantaged communities are classified as Quintile 1 schools, while those in the most economically advantaged communities are categorised as Quintile 5 [17]. Pupils in Quintiles 1 to 3 schools do not pay fees, and these schools are allocated more funding than schools in Quintiles 4 and 5 [18].

The ISHP is open to all the children in all the schools participating in the ISHP. All the children can access the full range of health services provided by the ISHP on-site (in-school) free of charge. The only condition that children have to fulfill in order to become eligible to participate is that they must present an informed consent form, that has been signed by their caregiver(s), to the school health professionals. The caregiver(s) could be a parent or grandparent, foster parent, aunt, uncle, older sibling, or any other person recognised as the legal caregiver.

A community-based non-profit organisation (NPO) with a very long history of working with these communities collaborated with the South African health and education departments (ministries) to implement the school health initiative in the communities where this study was conducted. It is noteworthy that the package of services provided in this initiative is larger than that provided in other schools where this partnership does not exist. This is because the NGO also developed partnerships with other service providers such as the dental health department of a local university. The private sector partnership also meant that school-going children receive donations of oral care and feminine hygiene products. Another element of this initiative is that it is open to all students in all participating schools whereas the standard programme provided by government in other schools is open to only Grade I of two primary schools.

The NPO provided school health services through trained school health nurses. The community health workers (CHW) affiliated with the NGOs have been providing health and social outreach services for over two decades. They do this with the help of volunteer community health workers who are co-ordinated by paid CHW supervisors [16]. Each supervisor is responsible for overseeing the work of CHWs in one or two of the thirteen communities where the NGO is active. The nurses visit various schools participating in the programme on days pre-negotiated with the schools once or twice yearly (depending on the availability of resources). They do so with their mobile clinics fully equipped with the requisite equipment, medical supplies, and human and other resources. Once in the schools, they provide free on-site healthcare services to the children regardless of their health status. In other words, all children who meet the criterion described earlier receive the whole package of services. The on-site services include immunisation, screening (assessment), health promotion and education, and treatment of minor conditions. With regard to screening, the school health nurses perform head-to-toe assessments for every child who has a signed informed consent form regardless of their health status. The aim of the screening intervention is to detect health conditions early for immediate treatment or referral to appropriate public health facilities, thereby reducing the burdens on children, parents, and the healthcare system.

Following the screening, all the children are provided with health education and health promoting items like toothpaste, toothbrushes, and sanitary pads, while those who have health conditions that are assessed as minor are treated on-site. Thereafter, children who require further assessment(s) or treatment(s) are referred to the public healthcare facilities. These could be a primary healthcare clinic or a hospital. The NGO, in addition to the referral letter provided by the school health nurse, documents each referred case and shares this information with the school. Thereafter, the school provides the child with an additional letter informing the caregiver of the health condition(s) diagnosed during the screening and inviting the caregivers to contact the NGO with their contact details, including their home address. If the caregiver follows up and provides this information, the NGO subsequently links them up with the supervisors of community health workers employed by the NGOs. Caregivers and their children are therefore assigned to CHWs already working in the specific communities where they live. NGO assists them in facilitating the follow-up with the public health facilities.

Gatekeeper permission was obtained from the relevant government departments, the NGO, and schools after we had received ethics approval from the Human Social Sciences Research Ethics Committee of the University of KwaZulu-Natal (Reference HSS/0570/016). For ethical reasons, we could not contact the children or their caregivers directly. We therefore leveraged the existing relationship between the caregivers and the community health workers affiliated to the NGOs. As we discuss later, the CHWs helped us facilitate community entry and the identification of potential participants.

### 2.1. Study Design

We utilised a qualitative research design to generate in-depth information about caregivers’ experiences accessing healthcare services for their school-going children. The design is suitable for this study as it allowed us to explore the lived experiences of caregivers, thereby enabling the researcher to capture the richness of their experiences regarding accessing healthcare services for their children [19]. In addition, this study employed the thematic analysis orientation described by Braun and Clarke [20].

### 2.2. Participants’ Characteristics and Selection Criteria

In order to recruit study participants, we developed a set of selection criteria to recruit study participants [21]. The selection criteria are as follows: a caregiver must be responsible for at least one primary-school-going child (Grade R—Grade 7) and be resident in one of the peri-urban communities where the study was conducted. We sought to include participants with various experiences relating to the research topic [21,22]. We, therefore, recruited participants belonging to three categories: (i) caregivers whose children were screened and treated on-site (in-school) and required no referrals for further assessment and treatment; (ii) caregivers whose children were screened and referred for further assessment and treatment, and who followed up by seeking treatment for their children; and (iii) caregivers who were referred to other facilities but did not seek further healthcare from the services where they were referred.

### 2.3. Sample Size

We followed Tolley and colleagues [23] (p. 60) in determining the sample size. The authors argue that small purposive samples are ideally suited to qualitative research. According to Tolley and colleagues [23] (p. 60), rather than aiming for large samples, qualitative researchers should aim to collect information representative of a range of experiences, perspectives, and behaviours relevant to the research question. Patton [24] (p. 185) argues that the logic of purposive sampling is different from the logic of probability sampling. Qualitative research scholars have also argued that qualitative researchers should aim to properly describe, justify, and explain their small sample size selection rather than seek to draw large samples. These, according to Patton [24], may not be useful for accomplishing the objectives of in-depth qualitative inquiries [23,24].

Following Patton [24] and Tolley and colleagues [23], we planned to recruit at least five participants for each of the three categories explained earlier. However, we could not reach the minimum number in two categories as we had hoped due to a number of constraints. As discussed earlier, the NGOs and the schools only have a list of children who have been referred to public health facilities and have to rely on the caregivers to provide their contact details before they could contact them to provide follow-up services. The NGO does not have the list or contact details of children who received on-site treatment only. It was, therefore, difficult to ascertain the exact number of children who accessed services on-site. In addition, many of the potential participants who provide their addresses to the NGO could not be reached using the details on file while others declined to participate. Yet others were not at home on the days and time that we had negotiated with them. 

The list of children who accessed follow-up services after they were referred was a more robust one because they had voluntarily followed up with the NGOs and were in more regular contact with the CHW supervisors in order to seek help in gaining access to the public health facilities. As a consequence, we were able to recruit more caregivers [9] from this category. But this was not the case for the other two categories. Children who were treated on-site (in-school) but were not referred for follow-up services were, in our estimation, the largest category. However, it was very difficult to find them because neither the NGOs nor the nurses specifically request their details after providing services since they dd not require any follow-up services. Nonetheless, the CHW supervisors used their community networks to locate a few caregivers that belong to this category and we were only able to recruit four caregivers that belong to this category. The third category are caregivers of children who had yet to access health services. Caregivers in this category did not follow up with the NGOs and were already not very responsive to the nurses even when they tried to follow up with them to facilitate access to the public healthcare system. We were only able to recruit four participants from this category. In total, we recruited seventeen caregivers who were available and willing to participate in the study.

### 2.4. Sampling Technique

Before the interviews, the school health nurses helped identify caregivers who met the selection criteria. This increased the opportunity to select information-rich participants [25]. A non-probability purposive sampling technique was then used to recruit the study participants. As discussed earlier, we adopted a purposive sampling strategy in selecting 17 participants who had the potential to provide in-depth information due to their previous experiences and availability to participate in the study [26]). The demographic characteristics of the participants are described in Table 1. The school health nurses informed the participants about the study telephonically and during their follow-up visits. The nurses also assisted with scheduling a convenient time for the interviews. Before the interviews, the nurses introduced the researchers to the participants.

### 2.5. Data Collection Procedure

Qualitative data for the study were collected using a semi-structured interview schedule that contained open-ended questions. The interview guide was developed based on relevant literature on access to healthcare services. It covered a range of themes, including demographic information of the caregivers and their children, health-seeking behavior, and caregivers’ experiences in accessing healthcare services. The interview guide was initially constructed in English and then translated into isiZulu.

Three research assistants, two females and a male, who were postgraduate (honours) students were recruited. They all had extensive experience in community-based work in a research setting and were all proficient in both isiZulu and English. Thereafter, they were trained by one of the authors (GBB) to help with data collection, transcription, and translation. The training was carried out in a workshop format and covered various themes: detailed information about the study context, study settings, and participants; interviewing skills; note taking; audio recording; documentation; and ethical issues. The interview schedule initially constructed in English was jointly translated into IsiZulu by the research assistants. These translations were also carried out in the same workshop after the training. Research assistants and the trainer had opportunities to debate issues before agreeing. This ensured that the translations were accurate. It also helped with uniformity in the research procedure.

Prior to the commencement of each interview, we requested and obtained informed consent from the caregivers to participate in the study. We also obtained their permission for the interviews to be audio-recorded. The research assistants conducted the interviews in isiZulu in the participants’ homes on days and times that we had earlier negotiated with the participants and nurses. They also obtained an audio-recording of each interview and took notes during the process. Each interview took between 30 to 45 min. All the participants provided consent to participate and for the interviews to be audio-recorded. GBB was present to provide oversight and to take additional notes during the interview sessions. All the interviews were transcribed verbatim, translated and back-translated where required by the research assistants.

### 2.6. Data Analysis

We analyzed the transcribed data following the five steps recommended by Braun and Clark [20] for thematic analysis. They include (i) familiarizing; (ii) generating initial codes; (iii) transforming the codes into emergent themes; (iv) reviewing all the themes across the entire data set; and (v) clustering the themes together in a meaningful way [21]. After coding the entire transcript, the themes were extracted, and a matrix was created using Excel software. The field notes further helped in analysing the data and understanding the themes. The data set was thoroughly reviewed to ensure that the data was meaningfully clustered under the appropriate themes. GBB conducted the initial analysis while OA reviewed all the themes. Both researchers reached an agreement after extensive discussions on the themes.

## 3. Results

Three themes emerged from our analysis of the qualitative data as shown in Table 2.

We discuss the three broad themes in the section that follows: (1) characteristics of the healthcare services; (2) caregivers’ perspectives about in-school health services; and (3) caregivers’ perspectives about follow-up services in the public health facilities.

### 3.1. Characterising the On-Site (In-School) Healthcare Services

Children are screened in-school for different health conditions by the school health nurses, and they identify health conditions that the caregivers were either unaware of or ignored. The caregivers’ accounts show that the children’s health conditions would have received little or no attention if they were not identified at school. Children in need of healthcare were either treated on-site or referred depending on the severity of the conditions. In making referrals, the nurses choose the health facility based on the nature of treatment and management that the child requires. The more severe conditions are referred to the hospitals that provide comprehensive health services. However, the majority of the children were referred to the primary healthcare clinics within the communities. In addition, the caregivers mentioned that the children sometimes accessed healthcare through mobile clinics that come to their communities. The NGO followed up with those that were referred, providing logistical and other kinds of supports. For example, they provided transportation to the referral sites for some of the children while others were taken to the health facilities by their caregivers.

### 3.2. Caregivers’ Perspectives about Health Services Provided On-Site (In-School)

Many caregivers mentioned they received letters to inform them about the screening and to obtain their consent for their children to be screened and treated.

*The principal called me and explained that people are coming to screen all the learners. So if you agree that your child should be screened, you need to sign an informed consent form which I signed. (Caregiver L—Primary School D).*

The supportive and caring role played by teachers and nurses in the in-school programme was a source of delight for some of the caregivers.

*I am happy with the way the teachers and the nurses treat the children in school, they become their parents when they are in school and are so concerned about the children’s health and entire well-being. Only nurses who love children like these ones do should be sent to schools (Caregiver K—Primary School D).*

The caregivers stated that the on-site services were helpful. According to them, the on-site (in-school) services relieves them of the cost of hospital or clinic visits, including for transportation and the payment for medications and treatments. In addition, the caregivers mentioned that the in-school services provide medications and other health-promoting items.

*The school programme is very helpful. They tell us about the illness and even refer the children to the hospital for more comprehensive treatments. The mobile clinic comes to examine the students; they also treat them and see the illnesses that we take for granted and those we parents wouldn’t have noticed. The treatments are very helpful. They even provide them with (sanitary pads) and other items. So I don’t have to buy pads; they also give them Colgate and toothbrushes (Caregiver I—Primary School A).*

They also noted that the treatment of health conditions had positive ramifications for the children and their learning abilities.

*The child had stomach ache in school. She was given medications in school and by the time she got back home she had stopped vomiting and she was fine (Caregiver K—Primary School D).*

*I asked my son about his eyes after he was taken to the hospital, he told me they are no longer painful and he can clearly see now. This time he is trying to do well in school as he is now in grade 5 (Caregiver C—Primary School B).*

Health education is a component of the school health package. The health education provided to the children in school helps them understand their health conditions, especially those living with HIV. The caregivers’ accounts showed that most of them found it challenging to educate children about their health conditions. This was especially the case for caregivers of children born with HIV. Many caregivers were concerned about the children’s reactions to the information, but the education provided in school made talking to the children about their health challenges easier. A caregiver narrated a situation she witnessed:

*There was this woman who was afraid to talk to her child about HIV. The child kept asking why she was using antiretroviral medications. She said they were told in school that HIV is transmitted through sexual intercourse, and she was not sexually active. The mother could not tell her that she was born with it. It’s really not easy, but she eventually got help from one of the school nurses. They both spoke to the child and explained to her that not all HIV positive people got infected through sexual intercourse (Caregiver N—Primary School D).*

### 3.3. Follow-Up after In-School Health Interventions

A few caregivers mentioned that they were informed about the results of the screening and referrals through phone calls. The CHW supervisors and teachers also visited the children and caregivers at their homes in some cases, especially when they needed to inform and educate the caregivers about the children’s health conditions and to monitor the child’s improvement.

*So after they screened him, they discovered a problem they then referred him for further treatment. They wrote me a letter after doing the assessments. They also wrote me a referral letter for the child. I could understand the content of the letter; I followed the instructions they gave in the referral letter (Caregiver L—Primary School D).*

*The CHW supervisor calls me, and she comes here sometimes to check on the child. She tries to monitor the treatment process and if the child is taking the medications correctly. She also asks if I’m noticing any improvement (Caregiver H—Primary School C).*

The caregivers further explained that NGO staffers and the clinic staffers regularly collaborate. According to them, this helps facilitate easier access to the primary healthcare clinic services. Some caregivers mentioned that they were able to avoid the long queues and reduce waiting time because they had referral letters from the school. Many of the caregivers also said that the services provided in-school motivates them to become actively involved in their children’s treatment process. The caregivers were happy that the school health nurses were concerned about their children’s welfare, and they expressed their gratitude:

*The programme makes life easier, especially for we mothers; it saves time because we sometimes skip the long queues at the clinic. I think it’s good because it means that the children are obtaining adequate care. The CHWs are so curious and concerned about the children’s wellbeing. The children are also attended to when the mobile clinics visit their communities. When the children arrive home with medication, even if the mother is lazy to go to the clinic but because the medication is there, they now assist their children (Caregiver L—Primary School D).*

However, some of the caregivers were not satisfied with the communication system. According to them, letters were the common means of information dissemination. These letters were sent through the children, who often forgot to deliver them to their caregivers, resulting in delays in seeking healthcare. A caregiver noted that:

*A letter was sent to inform us about the child’s condition, we were told to take him to the clinic, but the letter stayed for so long in the child’s school bag, because he forgot to deliver the letter. We could not take him to the clinic because we didn’t receive the letter until another letter was sent (Caregiver A—Primary School B).*

Another shortcoming of using letters as the main means of communication is that most caregivers have some challenges in understanding the information in the letters. These communication challenges contribute significantly to the delay in utilising healthcare services. A caregiver stated that:

*The school sent me letters, but I usually see them late; sometimes, when I check his (son’s) school bag. I think he even throws the letters away because he is so playful. I’m also not educated, so it’s difficult to read those letters (Caregiver O—Primary School D).*

Some of the caregivers’ accounts suggest that the referral system is still not adequately structured. They noted that the monitoring, follow-up, and feedback components need to be improved.

*They said I must take him to the hospital because the knee tumour can only be managed at the hospital, so they referred him to (a named district) hospital. So I took him to the hospital, and he had surgery, but I have not seen the school nurses since then (Caregiver E—Primary School A).*

### 3.4. Caregivers’ Perspectives about Accessing Follow-Up Services at Public Health Facilities

Access was described in terms of distance, cost of transportation, and waiting hours. Almost all the caregivers mentioned financial constraints as a major barrier to utilising healthcare services for their children. These financial challenges stem from a lack of jobs that will provide community members with an opportunity to earn an income, a common challenge in low-resource communities. The caregivers mentioned that they are willing to obtain quality healthcare services for their children but are constrained by a lack of finances. The distance to the public health facilities and the cost of transportation were the typical challenges described. According to them, some health facilities are far and hard to reach. Others described the difficulty they experienced in finding transportation to the health facilities to which they were referred. For example, a caregiver who resides in Community A and accesses treatment for her child with epilepsy at a facility 16 km away indicated that the taxi routes are inflexible, making hospital visits quite challenging. She also mentioned that she could not take the child for treatment for some months because she could not afford the cost of transportation which is approximately R60 ($3.71) for a round trip. As a result, her child could not access his monthly medications, and his epilepsy seizures became more frequent and severe.

*I found myself missing appointments because the hospital is far from here, and the taxis stopped going directly to the hospital. I sometimes do not have money for bus fares because I come from far and the transport fare is expensive. The transport fare cost R15 to the hospital and R15 back home for each of us… which amounts to R60 for each visit (Caregiver I—Primary School A).*

The challenge of distance and cost of transportation was also described by another caregiver, a grandmother responsible for eight grandchildren. The caregiver resides in Community C, and one of the children was referred to a clinic in another community. The distance between the two locations is about 26 km, and the cost of transportation is about R30 ($1.86) for each person per visit. The other four children were referred to the community clinic, and the transport fare is R20 ($1.25) for each of them. She also lamented that the transportation cost was high and unaffordable and that she could not take the children for the needed treatments for these reasons.

*I don’t have the transport fare to take the children for treatment. I’m unemployed. I depend on the grant and this ice cream I sell. It’s even difficult to feed them at times, so that’s why I’m unable to take them. It’s expensive because one was referred to a clinic which is far from here, and transportation costs about R50–60 for both of us, and the others were referred to the clinic here it will cost R20 for each person (Caregiver M—Primary School D).*

Another caregiver who had yet to access health services at a hospital for her children also mentioned that they are facing financial challenges as a result of unemployment:

*I plan to take both of them to the hospital when I get money, their father and I are out of jobs at the moment, but I will take them as soon as we get money; the transport fare cost about R30 for each person, it means R90 for the 3 of us…money is the problem (Caregiver A—Primary School B).*

On the other hand, some caregivers said that the health facilities (primary health centres) to which the children were referred are within the community. Regardless of this fact, they could not afford the cost of transportation.

*The clinic is not far if we take a taxi, but we cannot afford the taxi although it costs R7 ($0.44) for each person, so we usually walk, and it takes very long to get there and come back (Caregiver J—Primary School B).*

It is noteworthy that some caregivers were able to access follow-up care for their children with the help of the NGO. Nonetheless, they encountered other challenges at the health facilities.

Most of the caregivers indicated that they experienced long waiting hours at the clinic due to the large number of people served by these health facilities and the shortage of health professionals. Consequently, some of the caregivers were unable to access healthcare for their children.

*We wait for a long time, and I get exhausted. The queues are always very long, and I had to leave the clinic on a particular day because it didn’t look like they would attend to us after waiting for so long (Caregiver F—Primary School A).*

*It takes the whole day. We have to leave home at 5:45 am to catch the bus, to get there early because of the long queue, and we only come back home in the evening (Caregiver I—Primary School A).*

Another caregiver mentioned that adequate information about the structure and organisation of the facility is not provided, including information about the right queues to join. A grandmother noted:

*I will say it took time because in the hospital, especially when you’re old like me, you take time going around in circles, sometimes you wait and wait, and it is not comfortable moving along the benches, but I have to endure it because the child was sick (Caregiver E—Primary School A).*

Some caregivers described the health professionals’ attitudes, especially the nurses, as rude, unfriendly, and unhelpful, especially with regard to providing health education. They also mentioned that the health professionals do not provide adequate information about the children’s health conditions. For example, a caregiver whose child has asthma narrated her experience at a PHC.

*You can wait for 30 min while you have someone who is having an asthma attack. You wait 30 min while they are walking up and down. They don’t prioritise. Some of the staff are rude, but there are some warm ones. They do not provide adequate information. I once asked them what to do when my daughter has an attack when we are at home, and they responded by saying that there is nothing you can do when you are at home. They are impatient and sometimes give conflicting information on how to help the child. One will tell you that you need to pump the inhaler three times immediately and let her inhale at the count of 1 to 10 and at night do the same thing; another will tell you three times a day (Caregiver K—Primary School D).*

Despite the challenges that the caregivers confronted at the public health facilities, they were eventually able to access health services for their children. They expressed delight with the services they received and their children’s health outcomes.

*They (healthcare professionals) rendered high quality services. Series of tests were conducted at the clinic and in the hospital. The treatment helped my child; I’m happy (Caregiver B—Primary School B).*

*I would say I’m happy about the care and treatment they provided to the child when he was sick, the surgery helped a lot (Caregiver E—Primary School A).*

*The (epilepsy) seizures stopped for a long time when he started taking the medications they gave him at the hospital (Caregiver I—Primary School B)*

## 4. Discussion

This study reveals that caregivers in low-resource communities relied on the healthcare services provided in schools through the ISHP. Overall, the caregivers acknowledged and appreciate the role of the ISHP in providing an opportunity for their children to have access to healthcare services. They were grateful for the opportunity to have their children screened in the schools and for the health education provided by the school health nurses. According to the caregivers, the health education increased their knowledge of the conditions and enabled access to quality healthcare services for the children which would otherwise not be the case. They were also pleased that the school health initiative provided their children with items for health promotion like sanitary pads, toothbrushes, and toothpaste.

The screening programme provided the opportunity for the children’s conditions to be diagnosed and the caregivers felt this was very helpful. They were also delighted with the treatment that the children received on-site (in-school). The caregivers of children who received treatment on-site (in-school) described considerable improvements in their children’s health after receiving treatment.

Most of the caregivers appreciated the fact that their children were referred to other health facilities. They were pleased that the school health nurses and the NPO provided them with referral letters and followed up with them, in some instances providing transportation or collaborated with the primary healthcare facilities to facilitate their children’s access to healthcare, which were high points of the ISHP. Nonetheless, a few of the caregivers felt that there is some room for improvement, especially with regard to communication between the ISHP and the caregivers and follow-up after treatment in the public health facilities.

Caregivers reported financial constraints due to unemployment or low income. The cost of transportation to the public healthcare facilities is the major reason for financial stress among caregivers, resulting in delays in utilising healthcare. Previous studies show that people living in low-resource communities delay or find it difficult to use healthcare services due to financial constraints [15,27,28]. Delay or failure to use healthcare services for school-going children due to financial problems negatively impacts the children’s health, thereby affecting school attendance and learning capability. This undermines the goal of the school health initiative, which is to achieve holistic well-being for school-going children through the early detection and treatment of health conditions. The school health policy proffers a solution to this problem by making provisions for the transportation of children to the public healthcare facilities following a referral from the in-school health initiative. The Department of Social Development (DSD) is in charge of this arrangement [9]. However, this has yet to be implemented in practice, at least, in our study location. This finding highlights the need for the partnering departments to put mechanisms in place to monitor and ensure the proper implementation of the different components of the ISHP.

As the findings of this study show, caregivers experienced long waiting times and negative attitudes from the health professionals at the public health facilities, which discouraged the caregivers from using these services. The caregivers described a lack of trust in the healthcare system and perceived that limited value is placed on them and their children as service users. Previous studies conducted to evaluate the public health sector performances in South Africa show that long waiting times, noisy and crowded waiting areas, and the disrespectful behaviour of health professionals constitute major barriers to accessing and utilising healthcare services at government-owned healthcare facilities [28,29]. In addition, the findings of a systematic review conducted by Mannava and colleagues [30] revealed that health professionals’ attitudes to patients influence service utilisation and the perceived quality of care. Although the negative attitude of health workers relates to a broader systemic problem that should be addressed at the health systems level [31], these findings highlight the need to expand the range of services provided on-site (in-school), particularly in low-resource communities. Increasing the frequency of mobile clinic visits to low-resource communities and providing more sophisticated screening and treatment services through the mobile clinic could also reduce the number of children referred, especially for the less severe cases. 

Other factors that influenced service utilisation were the inadequate communication system adopted by the ISHP and the limited health information dissemination at the PHCs. Bernhardt [32] argues that communication, the starting point of the healthcare process, is a vital component of healthcare and can potentially influence service utilisation and health outcomes. Communication challenges could undermine the efforts of the school health nurses and the school health initiative as a whole. Therefore, the schools and the school health nurses should collaboratively explore better communication and information dissemination strategies in reaching out to caregivers. Increasing the number of home visits and strengthening follow-up services could facilitate effective communication. According to Olds and colleagues [32], consistent follow-up visits improve literacy levels, the relationship between the caregivers and nurses, and the children’s health and learning outcomes. Routine follow-up visits could also enhance the monitoring of the children’s health condition and provide opportunities to obtain feedback about the services accessed on-site and at the referral sites. Caregivers’ feedback is crucial to improving the quality and range of services provided and strengthening all the components of the ISHP.

The limitations of this study should be considered when interpreting the findings of this study. Firstly, the study provides the perspectives of the caregivers about factors influencing service utilisation for school-going children. Exploring the views of other stakeholders in the school health initiative could provide a deeper insight into issues relating to ISHP services utilisation. Secondly, we could not recruit an equal number of participants for all the categories as only a few caregivers that met the inclusion criteria were available to participate in the study. However, this challenge was addressed by including caregivers of children from different schools and community settings across all the categories to obtain a range that represents different perspectives. Thirdly, the fact that the study was conducted in only three low-resource communities in KwaZulu-Natal might affect the transferability of the study to other contexts.

## 5. Conclusions

Caregivers found the ISHP very useful in the detection and treatment of the health conditions for their school-going children. Logistical challenges with the follow-up with caregivers highlight the need for innovative community-based health promotion and health communication strategies. In addition, there is a need for the government departments to fully implement the various elements of the school health policy. This policy makes provisions for addressing some of the challenges that we identified in our study such as transportation and other services to facilitate access to public health facilities. This could help improve the effectiveness of the ISHP and help achieve its utmost goal, which is to improve the general health of school-going children and address barriers to learning to improve educational outcomes.

## Figures and Tables

**Table 1:** Participants’ characteristics.

Gender	Frequency	Percentage
Male	1	5.88
Female	16	94.12
Total	17	
Age		
* 20–29	5	29.35
30–39	4	23.53
40–49	3	17.70
50–59	3	17.70
>60	2	11.76
Schools (Quintiles)		
School A (2)	6	35.29
School B (3)	4	23.53
School C (2)	3	17.65
School D (2)	4	23.53
Relationship with children		
Mother	8	47.05
Grandmother	5	35.31
Aunt	2	11.76
Stepmother	1	5.88
Father	1	5.88
Employment status		
Unemployed	7	41.18
Employed	4	23.53
Self-employed	3	17.65
Grant receiver	2	11.76
Pensioner	1	5.88
Total	17	

* The youngest caregiver was 27 years old.

**Table 2:** Themes relating to the perspectives of caregivers about in-school services and follow-up services, and access to public health facilities.

Super-Theme	Themes
Perspectives of caregivers about in-school, follow-up, and access to public health facility services	Characterising the on-site (in-school) healthcare services Supportive role of teachers and school health nurses; Relief from the financial and logistical burden of accessing healthcare for children; Positive health outcomes for children; Important role of health education.
	Caregivers’ perspectives about health services provided on-site (in-school) Follow-up to in-school screening by school and NGO; Role of collaboration between NGO and PHC clinics in access and adherence to medication; Challenges with communication and follow-up from schools and NGOs.
	Caregivers’ perspectives about follow-up activities (formal public health facilities) Cost of transportation; Wait times at public health facilities; Navigating the large and complex health facilities; Attitude of health professionals at public hospitals; Satisfaction with health services and health outcomes.

## Data Availability

The data that support the findings of this study are available upon request from the corresponding author, G.B.B.
